# Explainable machine learning–based prediction of postoperative pulmonary infection after aneurysmal subarachnoid hemorrhage clipping and exploratory analysis of enteral nutrition timing: a dual-cohort retrospective study

**DOI:** 10.3389/fneur.2026.1873566

**Published:** 2026-10-08

**Authors:** Lin Li, Xiang Li, Shufang Shi

**Affiliations:** 1 Department of Nursing, Shanxi Bethune Hospital, Shanxi Academy of Medical Sciences, Third Hospital of Shanxi Medical University, Tongji Shanxi Hospital, Taiyuan, China; 2 Department of Neurosurgery, Shanxi Bethune Hospital, Shanxi Academy of Medical Sciences, Third Hospital of Shanxi Medical University, Tongji Shanxi Hospital, Taiyuan, China

**Keywords:** aneurysmal subarachnoid hemorrhage, clinical prediction model, enteral nutrition, explainable machine learning, neurocritical care nursing, postoperative pulmonary infection, precision nursing

## Abstract

**Background:**

Postoperative pulmonary infection (PPI) is a common complication after microsurgical clipping for aneurysmal subarachnoid hemorrhage (aSAH) and is associated with impaired recovery and greater use of hospital resources.

**Objective:**

To develop and temporally validate an explainable machine-learning model for PPI risk stratification and to explore the association between enteral nutrition (EN) initiation timing and subsequent PPI risk.

**Methods:**

This dual-cohort retrospective study was reported in accordance with Transparent Reporting of a multivariable prediction model for Individual Prognosis Or Diagnosis plus Artificial Intelligence (TRIPOD+AI) principles. Candidate predictors were selected using a combined least absolute shrinkage and selection operator (LASSO) regression and random-forest importance strategy. Nine machine-learning algorithms were developed and compared in terms of discrimination, calibration, and clinical utility using decision-curve analysis.

**Results:**

In the validation cohort, the Naive Bayes model showed the best overall performance [area under the receiver operating characteristic curve (AUC), 0.811; 95% confidence interval (CI), 0.755–0.855]. SHAP analysis identified the major contributors to the primary 24-h prediction model. Separately, a secondary exploratory analysis of continuous EN initiation timing identified a nonlinear pattern, with a bootstrap median model-derived inflection point of 24.0 h (95% bootstrap interval, 23.0–27.0 h).

**Conclusions:**

This explainable model may support early PPI risk stratification after aSAH clipping. The observed EN timing signal should be interpreted as exploratory and warrants prospective validation before clinical implementation.

## 1 Introduction

Aneurysmal subarachnoid hemorrhage (aSAH)—defined as spontaneous bleeding into the subarachnoid space following the rupture of an intracranial aneurysm—remains one of the most devastating neurological emergencies (1, 2). The condition is characterized by an abrupt onset, profound morbidity, and a crude global incidence of approximately 7.9 per 100,000 person-years (3). The severity of this burden is underscored by the 2023 American Heart Association/American Stroke Association (AHA/ASA) guidelines, which report persistently high prehospital (22–26%) and inpatient (13–20%) mortality rates (4). For survivors, the trajectory of neurological recovery is frequently derailed by severe secondary complications, placing an immense demand on precision neurocritical care.

Early aneurysm securement is central to the acute management of aSAH and is achieved predominantly by microsurgical clipping or endovascular coiling (4). Current guidelines recommend that treatment modality be individualized according to patient and aneurysm characteristics and the likelihood of achieving complete aneurysm obliteration (4). Postoperative pneumonia may occur after either treatment strategy. In a retrospective cohort of 843 patients with aSAH, postoperative pneumonia occurred in 114 of 429 patients (26.57%) undergoing endovascular coiling and 143 of 414 patients (34.54%) undergoing surgical clipping (*p* = 0.015) (5). Because treatment allocation was nonrandom and influenced by clinical and aneurysm-related characteristics, this difference should be interpreted descriptively rather than as evidence of a causal effect of treatment modality. Nevertheless, the substantial burden of postoperative pneumonia among patients undergoing microsurgical clipping underscores the clinical importance of early risk stratification and preventive care in this population.

Patients undergoing microsurgical clipping may require intensive postoperative monitoring and, depending on neurological and clinical status, sedation, mechanical ventilation, and prolonged immobilization, all of which may contribute to vulnerability to secondary complications. Postoperative pulmonary infection (PPI) is among the most frequent and clinically consequential complications after microsurgical clipping for aSAH. Its determinants are multifactorial and dynamic, involving neurological severity, airway instrumentation and mechanical ventilation, impaired airway protection (e.g., dysphagia and aspiration), and nutritional and other care-related factors, including the timing of enteral nutrition (EN) initiation (6, 7). PPI is associated with impaired oxygenation, greater need for ventilatory support, prolonged intensive care unit (ICU) and hospital stays, increased healthcare resource use, and unfavorable neurological recovery (8). Given its substantial incidence and clinical burden, PPI represents an important target for early risk stratification and preventive management.

Existing prediction studies in this field are predominantly retrospective, based on moderate sample sizes, often include heterogeneous treatment strategies, and frequently emphasize discrimination, typically quantified by the area under the receiver operating characteristic curve (AUC), while providing limited assessment of external validity, calibration, clinical utility, and model interpretability (9, 10). Machine learning (ML) provides a framework for capturing nonlinear relationships and interactions in perioperative data, whereas explainable approaches such as SHapley Additive exPlanations (SHAP) can improve transparency by quantifying the contribution of individual predictors to model outputs (10–12).

The primary aim of this study was to develop and temporally validate an explainable model for early PPI risk stratification in patients with aSAH undergoing microsurgical clipping, using predictors available at the predefined prediction time point. The secondary aim was to explore potential nonlinear associations between EN initiation timing and subsequent PPI risk.

## 2 Materials and methods

### 2.1 Study setting and participants

This study was reported in accordance with Transparent Reporting of a multivariable prediction model for Individual Prognosis Or Diagnosis plus Artificial Intelligence (TRIPOD+AI) principles (13). Data were collected from two independent cohorts: a training cohort used for model development and an independent validation cohort used for performance evaluation. Aneurysm location was abstracted from the available clinical records and classified according to circulation territory as anterior or posterior circulation, which represented the highest level of anatomical detail consistently available across the cohort. Specific artery-level locations were not systematically recorded across the entire cohort and therefore could not be reported reliably. To minimize temporal information leakage and enhance clinical usability, we prespecified a postoperative 24-h landmark as the prediction time point. All predictors were strictly restricted to data available within the first 24 postoperative hours. For instance, mechanical ventilation was coded as a binary indicator of whether the patient was on a ventilator at the 24-h landmark, rather than total ventilation duration. Similarly, for the primary model, EN was coded as a binary indicator of whether EN had been initiated by the 24-h landmark. Continuous EN initiation timing was reserved for a separate secondary exploratory analysis and was not included as a predictor in the primary 24-h landmark model. All variables that could only be determined after the 24-h landmark were explicitly excluded from the predictor set. This approach ensured that predictors in the primary model were available at the time of risk assessment and minimized the risk of temporal information leakage from future events.

Initial data cleaning involved the removal of non-clinical identifiers (e.g., patient names, IDs). We reported the proportion of missing data for each candidate predictor. All candidate predictors had complete data with no missing values in both the development and validation cohorts; therefore, no imputation was performed. This was confirmed by systematic examination of completeness for each variable, ensuring consistent data quality across both datasets. Binary variables were coded as 0/1 and nominal variables were one-hot encoded. Scaling parameters for continuous variables were estimated in the development cohort and then applied unchanged to the validation cohort. To ensure comparable scales across different clinical features, numerical variables were normalized using StandardScaler (μ = 0, σ = 1) before being fed into linear and distance-based models.

### 2.2 Data collection and preprocessing

This study was a retrospective observational cohort study conducted in the Shanxi Bethune Hospital. Patients admitted between January 2020 and March 2025 with a diagnosis of aSAH who underwent microsurgical aneurysm clipping were enrolled. Participants were classified into an infection group and a non-infection group according to whether they developed PPI. PPI was diagnosed based on a combination of clinical, radiological, and microbiological criteria, including new or progressive infiltrates on chest imaging plus at least two of the following: fever (>38 °C), leukocytosis or leukopenia, purulent sputum, or positive microbiological cultures (14). EN initiation timing was defined as the time interval (in hours) from the completion of aneurysm clipping surgery to the first documented administration of any enteral feeding product via the enteral route (nasogastric or nasointestinal tube). The decision to initiate EN, the choice of feeding route, and the selection of enteral formula were made by the attending neurointensivists and clinical dietitians according to standard institutional protocols. EN was typically initiated when hemodynamic stability was achieved and there were no absolute contraindications (e.g., gastrointestinal bleeding, ileus, or unstable bowel obstruction). The exact time of first EN administration was extracted from electronic nursing records and confirmed by crossreferencing with the enteral nutrition administration flow sheets. The inclusion criteria were as follows: (1) aSAH confirmed by high-quality vascular imaging, including computed tomography angiography (CTA), magnetic resonance angiography (MRA), or digital subtraction angiography (DSA); (2) treatment with craniotomy and aneurysm clipping; (3) age ≥18 years; and (4) length of hospital stay ≥48 h. The exclusion criteria were: (1) PPI related to the index disease diagnosed prior to admission that had not resolved after admission; (2) presence of other severe physical or psychiatric disorders; and (3) incomplete data collection. The primary outcome was the first episode of PPI occurring after the prespecified prediction time point and before hospital discharge.

### 2.3 Feature selection strategy

To prevent overfitting and identify the most parsimonious set of predictors, we employed a dual-track feature selection approach on the training cohort:

Lasso Regression (L1 Regularization): the Least Absolute Shrinkage and Selection Operator (Lasso) with 5-fold cross-validation was used to shrink the coefficients of redundant variables to zero.

Random Forest Importance: a Random Forest classifier (100 estimators) was utilized to rank features based on their Gini importance, capturing complex non-linear associations.

The union of features identified by both methods was selected as the final input set for model construction, ensuring that both linear and non-linear contributing factors were preserved.

### 2.4 Machine learning algorithms and model development

Nine diverse machine learning algorithms were implemented and compared to identify the optimal predictive architecture:

Linear/Statistical Models: logistic Regression, Naive Bayes.

Ensemble Models: random Forest, Extreme Gradient Boosting (XGBoost),Light Gradient Boosting Machine (LightGBM).

Tree-based Model: decision Tree.

Advanced Models: support Vector Machine (SVM), Multi-Layer Perceptron (MLP) Neural Network, and K-Nearest Neighbors (KNN).

To address the imbalance in the target variable (infection vs. non-infection), we applied class-weight balancing or adjusted the scale_pos_weight parameter for ensemble models, assigning higher penalties to the misclassification of the minority class (infected cases).

### 2.5 Model evaluation and validation

Model performance was rigorously evaluated on the independent validation cohort using three dimensions:

Discrimination: measured by the Area Under the Receiver Operating Characteristic curve (AUC-ROC). The 95% confidence intervals (CI) for AUC were calculated using bootstrapping (1,000 iterations). Accuracy, sensitivity, and specificity were also calculated.

Calibration: model performance was evaluated in terms of discrimination, calibration, and clinical utility. We report the AUC with 95% confidence intervals, sensitivity, specificity, and accuracy, and we assessed calibration graphically together with decision-curve analysis.

Clinical Utility: evaluated through decision curve analysis (DCA), which quantifies the net benefit provided by the model across various threshold probabilities.

### 2.6 Model interpretability (SHAP analysis)

To transition from a “black-box” prediction to clinical transparency, we employed SHAP based on cooperative game theory.

Global Interpretation: a SHAP beeswarm plot was generated to rank feature importance and visualize the direction of impact (positive or negative) of each variable on the infection risk.

Local Interpretation: waterfall and Force plots were used to provide individualized risk explanations for specific high-risk cases.

Exploratory Dependence Analysis: sHAP dependence plots were constructed to investigate the non-linear relationship and potential “threshold effects” of key clinical variables, such as the timing of enteral nutrition initiation.

For the secondary exploratory analysis, continuous EN initiation timing was evaluated in a separate exploratory Naive Bayes model and was not included in the primary 24-h landmark model used for model-performance estimation. SHAP dependence analysis was used to characterize potential nonlinear patterns between continuous EN initiation timing and model output. A locally weighted scatterplot smoothing (LOWESS) curve was fitted to patient-level SHAP values to provide a descriptive representation of the overall trend. The approximate zero-crossing of the smoothed SHAP trajectory was considered an exploratory model-derived inflection point. To assess the internal stability of the estimated inflection location, the LOWESS fitting and zero-crossing estimation were repeated across 1,000 bootstrap resamples. The resulting bootstrap distribution was summarized using its median and 95% percentile interval. This analysis was exploratory and was not intended to establish a causal effect, clinically validated cutoff, or optimal intervention time.

### 2.7 Statistical software

All data processing, model training, and visualization were performed using Python (version 3.x). Key libraries included scikit-learn for general machine learning, xgboost and lightgbm for gradient boosting, matplotlib and seaborn for visualization, and the shap library for model interpretability.

### 2.8 Ethics approval

The study protocol was approved by the Institutional Review Board (IRB) of the Shanxi Bethune Hospital (Approval No. YXLL-2024-160). The study was conducted in accordance with the ethical principles of the Declaration of Helsinki.

## 3 Results

### 3.1 Patient characteristics

A total of 618 consecutive patients were screened for eligibility. After excluding 29 patients with pre-existing or unresolved pulmonary infection, 38 patients with incomplete outcome data, and 34 patients meeting other exclusion criteria, 517 patients were included in the final analysis. Of these, 397 patients formed the development cohort and 120 patients formed the validation cohort. Overall, 191 of 517 patients (36.9%) developed PPI, including 153 of 397 (38.5%) in the development cohort and 38 of 120 (31.7%) in the validation cohort.

Baseline characteristics of patients in both cohorts are summarized in Table 1. The median age was 57.0 years [interquartile range (IQR), 49.0–63.0] in the training cohort and 58.0 years (IQR 50.0–64.0) in the validation cohort. Female patients accounted for 54.4% and 54.2% of the training and validation cohorts, respectively. There were no statistically significant differences between the two cohorts in most baseline variables, indicating good comparability. However, several variables showed statistically significant differences, including stroke history (*p* = 0.032), albumin levels (*p* = 0.006), operation time (*p* < 0.001), and nasointestinal tube use (*p* = 0.018). Continuous variables were compared using the Mann–Whitney U test, while categorical variables were analyzed using the χ^2^ test or Fisher's exact test, as appropriate. The number of outcome events was considered sufficient for model development, although the results should still be interpreted in light of the single-center design and the sample size of the validation cohort.

**Table 1:** Baseline characteristics of the development and validation cohorts.

Characteristic	Training cohort (*n* = 397)	Validation cohort (*n* = 120)	*P* value
Female gender, *n* (%)	216 (54.4)	65 (54.2)	1.000
Age (years), Median (IQR)	57.0 (49.0–63.0)	58.0 (50.0–64.0)	0.388
Height (cm), Median (IQR)	165.0 (160.0–175.0)	165.5 (160.0–175.0)	0.319
Weight (kg), Median (IQR)	65.0 (60.0–75.0)	65.5 (62.5–76.0)	0.118
Stroke history, *n* (%)	24 (6.0)	15 (12.5)	0.032
Heart disease, *n* (%)	21 (5.3)	4 (3.3)	0.524
Hypertension, *n* (%)	183 (46.2)	63 (52.5)	0.270
Diabetes mellitus, *n* (%)	16 (4.0)	6 (5.0)	0.825
COPD, *n* (%)	6 (1.5)	1 (0.8)	1.000
Current smoker, *n* (%)	146 (36.8)	43 (35.8)	0.936
Alcohol consumption, *n* (%)	93 (23.5)	23 (19.2)	0.385
Albumin (g/L), Median (IQR)	36.6 (33.4–39.7)	38.0 (35.2–41.5)	0.006
Platelets ( × 10^9^/L), Median (IQR)	190.0 (154.0–232.0)	197.0 (158.8–239.2)	0.369
WBC count ( × 10^9^/L), Median (IQR)	11.6 (8.6–15.2)	12.0 (9.1–14.8)	0.865
Neutrophils ( × 10^9^/L), Median (IQR)	9.9 (7.0–13.4)	10.6 (6.8–13.9)	0.701
Lymphocytes ( × 10^9^/L), Median (IQR)	0.9 (0.7–1.5)	1.0 (0.7–1.4)	1.000
Potassium (mmol/L), Median (IQR)	3.8 (3.5–4.0)	3.8 (3.5–4.0)	0.803
Sodium (mmol/L), Median (IQR)	139.0 (137.1–141.1)	138.8 (136.7–140.9)	0.274
GCS score, Median (IQR)	14.0 (7.0–14.0)	14.0 (8.0–15.0)	0.438
Blood loss (ml), Median (IQR)	300.0 (200.0–400.0)	300.0 (200.0–400.0)	0.921
Hunt–HESS grade, *n* (%)	99 (24.9)	31 (25.8)	0.938
Aneurysm circulation territory, *n* (%)			1.000
Anterior circulation	280 (70.5)	85 (70.8)	
Posterior circulation	117 (29.5)	35 (29.2)	
Aneurysm size, *n* (%)	308 (77.6)	89 (74.2)	0.514
Emergency surgery, *n* (%)	254 (64.0)	68 (56.7)	0.180
Operation time (h), Median (IQR)	5.5 (4.0–7.0)	4.0 (3.5–5.1)	< 0.001
Tracheal intubation, *n* (%)	256 (64.5)	75 (62.5)	0.773
Tracheostomy, *n* (%)	116 (29.2)	35 (29.4)	1.000
Mechanical ventilation, *n* (%)	179 (45.1)	46 (38.3)	0.229
EN initiation timing (h), Median (IQR)	9.0 (6.0–24.0)	8.0 (0.0–27.5)	0.373
Nasointestinal tube, *n* (%)	9 (2.3)	9 (7.5)	0.018
Nasogastric tube, *n* (%)	262 (66.0)	77 (64.2)	0.795
PPI, *n* (%)	153 (38.5)	38 (31.7)	0.208

Continuous variables are presented as median (IQR) and categorical variables as n (%).

### 3.2 Feature selection and variable identification

PPI was defined using a structured institutional adjudication framework based on compatible clinical, radiographic, and microbiological criteria: new or progressive pulmonary infiltrates on chest imaging, together with at least two of the following findings: fever (>38 °C), abnormal leukocyte count, purulent respiratory secretions, or positive respiratory microbiology. For patients with underlying cardiopulmonary disease, radiographic persistence was required to reduce outcome misclassification. Outcome adjudication was performed by two clinicians who were blinded to model predictions, with disagreements resolved by consensus. To identify the most robust predictors for PPI, a dual-track feature selection strategy was employed. First, Lasso regression was used to shrink redundant variable coefficients to zero, retaining variables with the highest linear predictive power (Figure 1A). Simultaneously, Random Forest (RF) importance ranking was conducted to capture non-linear contributions, identifying the top 15 features based on Gini importance (Figure 1B).

**Figure 1:**
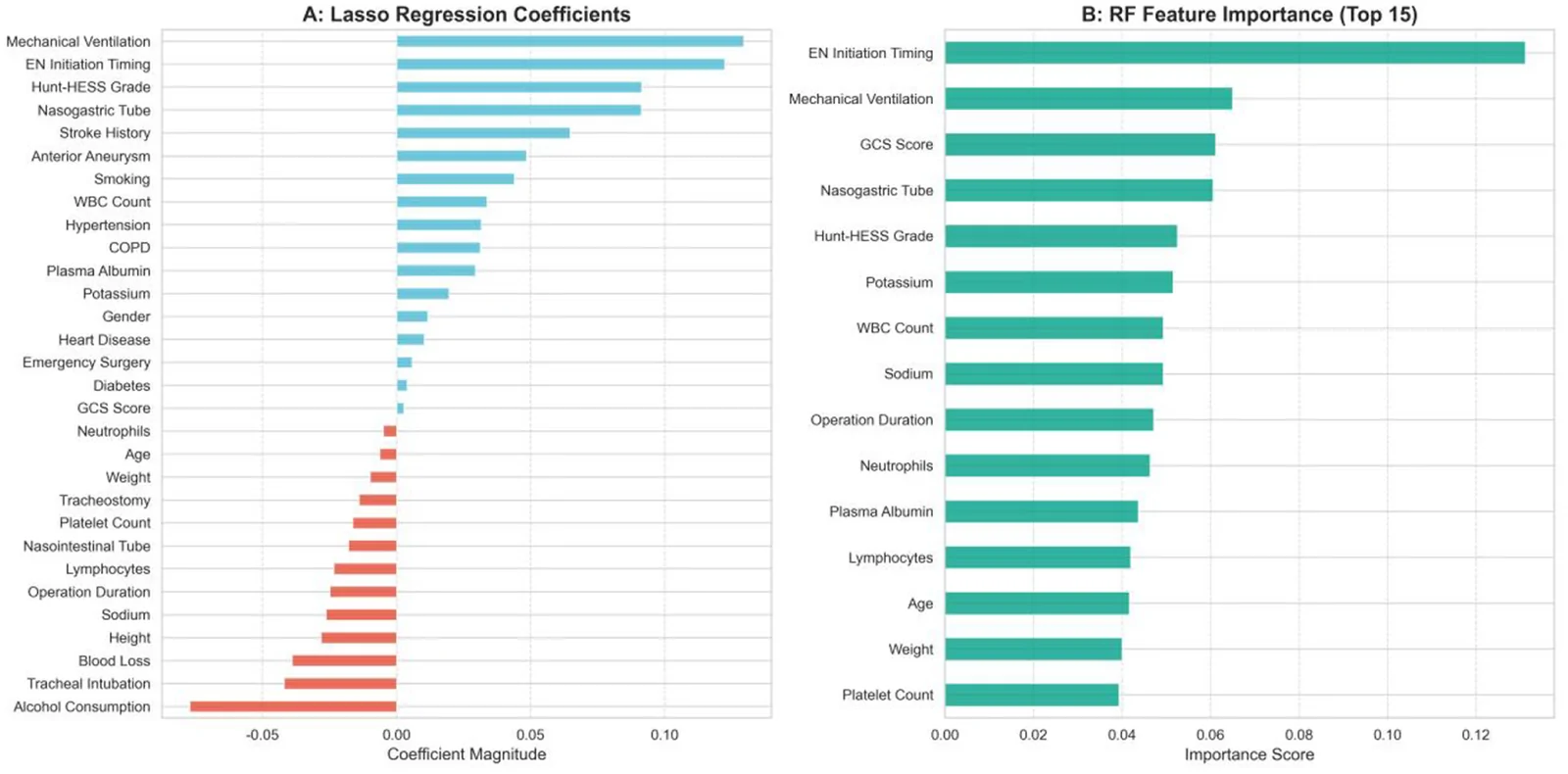
Variable Selection Process. **(A)** Lasso regression coefficient profiles. Each bar represents the coefficient magnitude of a clinical feature; blue indicates a positive correlation with PPI, while orange indicates a negative correlation. **(B)** Feature importance ranking from the Random Forest model. Features are ordered by their contribution to the model's predictive accuracy.In the primary prediction model, the EN variable represents EN initiation status by the 24-h landmark; continuous EN initiation timing was not included as a primary predictor.

The union of these two methods resulted in the final inclusion of 15 variables. Notably, Mechanical Ventilation, EN initiation status by the 24-h landmark, and Stroke History were consistently identified as top predictors across both algorithms. Other significant variables included Glasgow Coma Scale (GCS) score, Nasogastric tube use, and Hunt-HESS grade. The number of candidate predictors was restricted relative to the number of outcome events to reduce the risk of overfitting.

### 3.3 Model performance and comparison

Nine machine learning algorithms were trained and evaluated on the independent validation cohort. Among them, the Naive Bayes model demonstrated the superior discriminative ability with an AUC of 0.811 [95% CI: (0.755-−0.855)], followed by the MLP (AUC = 0.797) and LightGBM (AUC = 0.751) (Figure 2A). Detailed performance metrics, including sensitivity, specificity, and accuracy, are summarized in Table 2.

**Figure 2:**
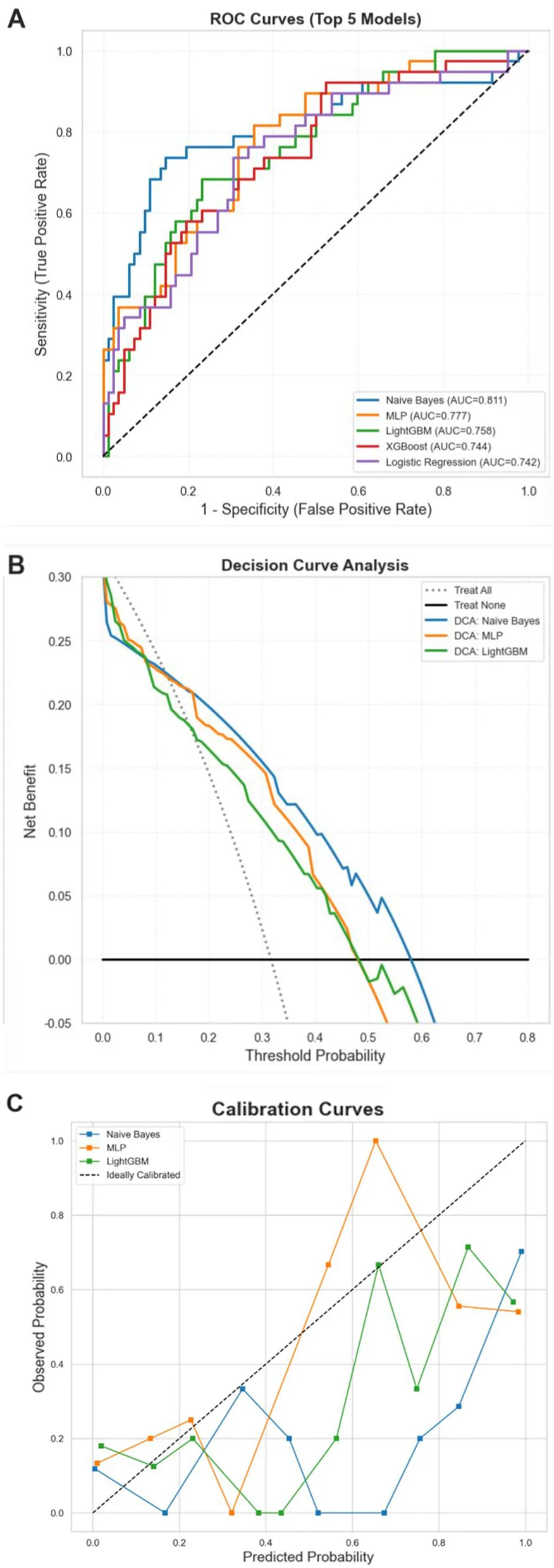
Performance Evaluation in the Validation Cohort. **(A)** Receiver Operating Characteristic (ROC) curves for the top 5 performing models, with Naive Bayes achieving the highest AUC (0.811). **(B)** Decision Curve Analysis (DCA) demonstrating the clinical net benefit of using the predictive models across various threshold probabilities. **(C)** Calibration curves showing the agreement between predicted probabilities and actual observed infection rates.

**Table 2:** Performance of the machine-learning models in 524 the validation cohort.

Model	AUC	95% CI	Sensitivity	Specificity	Accuracy
Naive Bayes	0.811	0.755–0.855	0.763	0.720	0.733
MLP	0.797	0.796–0.853	0.684	0.744	0.725
LightGBM	0.751	0.742–0.798	0.711	0.707	0.708
Logistic Regression	0.742	0.729–0.767	0.737	0.671	0.692
XGBoost	0.719	0.717–0.787	0.658	0.659	0.658
SVM	0.718	0.728–0.774	0.579	0.756	0.700
KNN	0.699	0.715–0.759	0.447	0.707	0.625
Random Forest	0.689	0.674–0.754	0.579	0.744	0.692
Decision Tree	0.638	0.638–0.665	0.605	0.683	0.658

The DCA further confirmed the clinical utility of the models. The Naive Bayes and MLP models provided a higher net benefit compared to the “treat-all” or “treat-none” strategies across a wide range of threshold probabilities (approximately 0.10–0.60) (Figure 2B). While the calibration curves showed some fluctuations due to the validation cohort's sample size, they generally maintained a consistent trend between predicted and observed probabilities (Figure 2C).

### 3.4 Model interpretation via SHAP

SHAP (SHapley Additive exPlanations) was applied to the best-performing model (Naive Bayes) to provide a transparent interpretation of its decision-making process. The SHAP summary plot revealed that Stroke History had the greatest overall impact on the model's output, with positive values indicating an increased risk of infection. Similarly, EN initiation status by the 24-h landmark, use of a nasogastric tube, and mechanical ventilation were positively associated with infection risk (Figure 3A). Individual-level explanation (Waterfall plot) for a high-risk patient (No. 34) demonstrated how specific clinical factors, such as Chronic obstructive pulmonary disease (COPD), dramatically pushed the risk score toward infection (SHAP value +0.85) (Figure 3B). The corresponding SHAP force plot provided a complementary visualization of the direction and magnitude of feature contributions for the same patient (Figure 3C).

**Figure 3:**
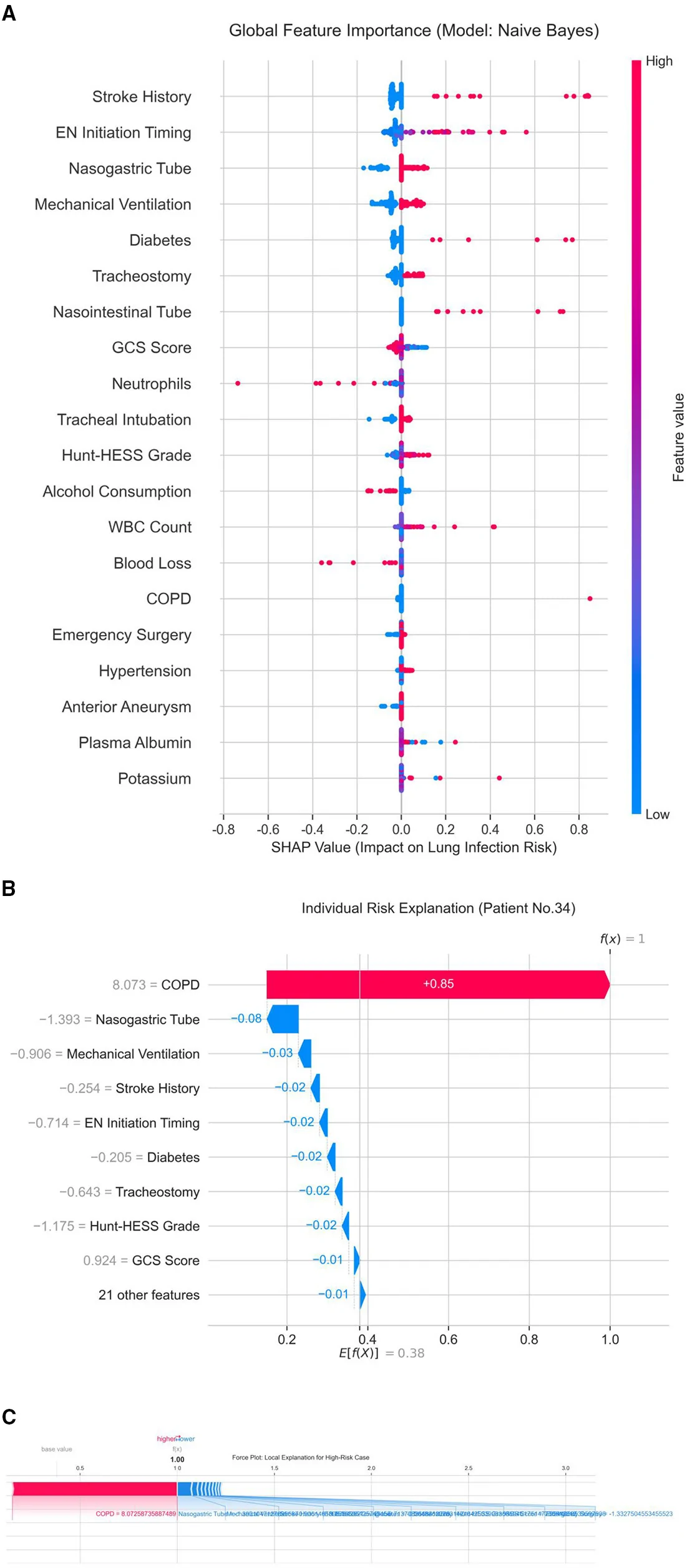
Global and Local Model Interpretability (SHAP). **(A)** SHAP beeswarm plot illustrating the distribution of the impact each feature has on the model's prediction. Each dot represents one patient; red indicates a higher value of the feature, and blue indicates a lower value. **(B)** SHAP waterfall plot for an individual high-risk case (Patient No. 34), showing the additive contribution of each clinical feature to the final risk score. **(C)** SHAP force plot providing a compact visualization of the local explanation for the same high-risk patient. In the primary prediction model, the EN variable represents EN initiation status by the 24-h landmark; continuous EN initiation timing was not included as a primary predictor.

Secondary exploratory analysis of EN initiation timing. Continuous EN initiation timing was not included in the primary 24-h landmark model and was evaluated separately as a secondary exploratory analysis. The SHAP dependence plot illustrated the relationship between continuous EN initiation timing and its contribution to model output, with operation duration represented by the color gradient (Figure 4). Further analysis using LOWESS smoothing suggested a nonlinear relationship between continuous EN initiation timing and model output (Figure 5). The LOWESS-smoothed SHAP trajectory crossed the zero baseline at approximately 25.0 h. Across 1,000 bootstrap resamples, the median estimated model-derived inflection point was 24.0 h, with a 95% bootstrap percentile interval of 23.0–27.0 h, suggesting internal stability in the location of this exploratory signal. Earlier EN initiation tended to correspond to negative SHAP contributions, whereas later initiation tended to correspond to positive SHAP contributions. Because SHAP values reflect contributions to model predictions rather than causal effects, the approximately 24–25-h region should be interpreted as a hypothesis-generating model-derived pattern rather than a clinically validated intervention threshold. These findings should be interpreted as exploratory and hypothesis-generating, warranting prospective validation.

**Figure 4:**
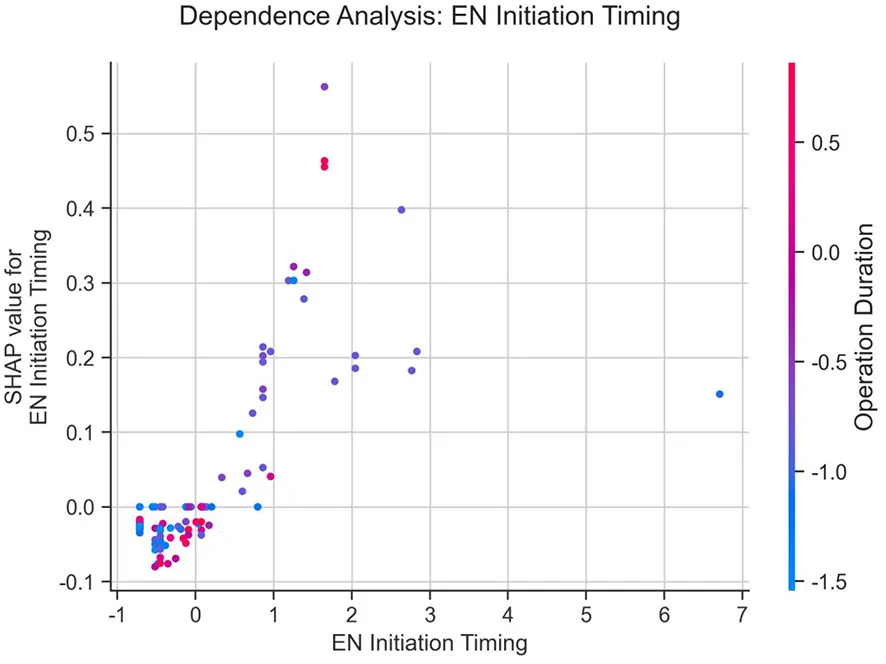
SHAP Dependence Plot for EN Initiation Timing. Scatter plot showing the relationship between the timing of enteral nutrition initiation (x-axis) and its SHAP value (y-axis). The vertical dispersion of points at specific time intervals suggests a threshold effect where delayed initiation significantly elevates infection risk scores.

**Figure 5:**
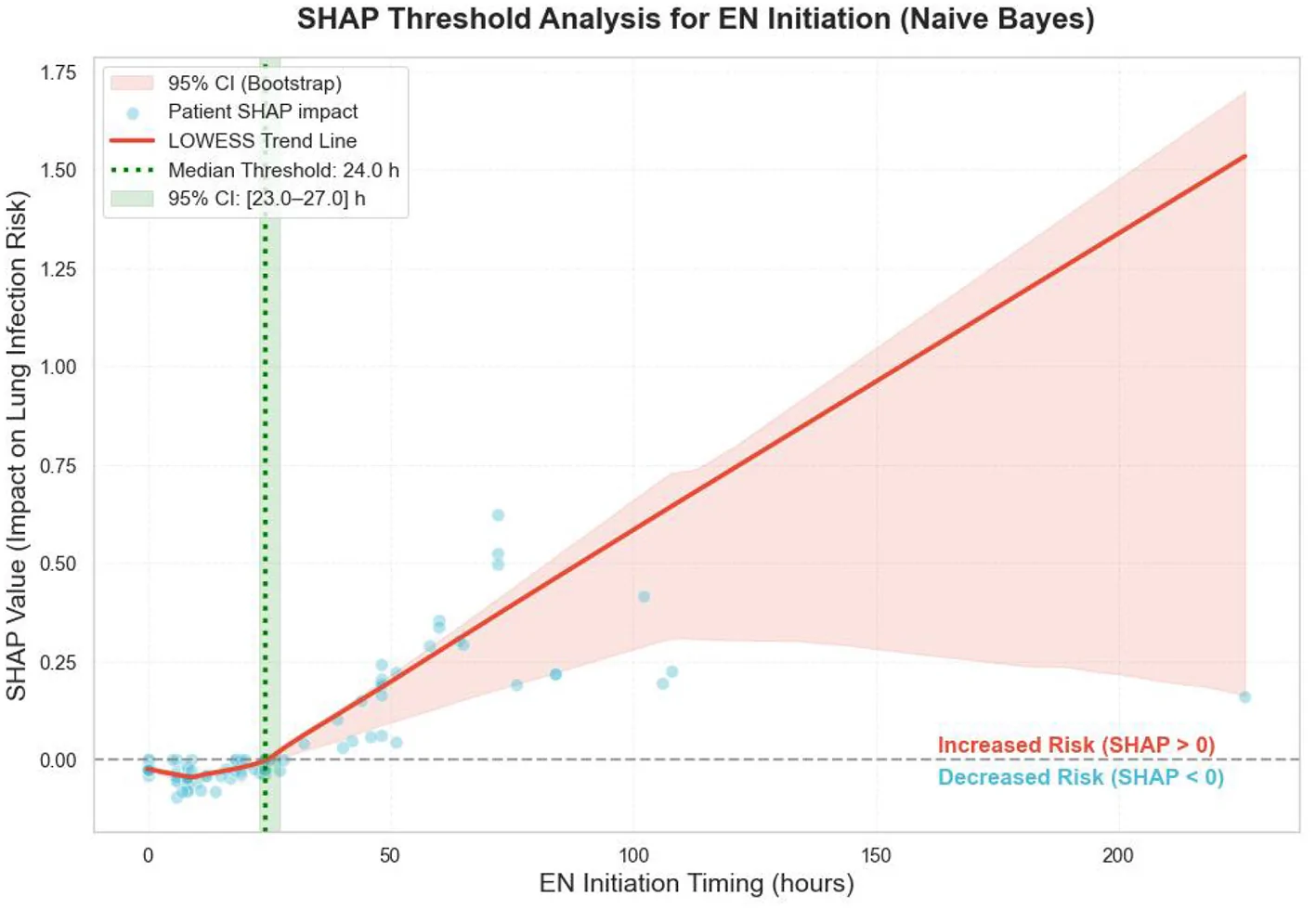
SHAP dependence plot illustrating the non-linear impact of enteral nutrition (EN) initiation timing on PPI risk. The blue scatter points represent individual patient SHAP values derived from the Naive Bayes model, reflecting the specific impact of EN timing on each patient's risk. The solid red line represents the LOWESS (Locally Weighted Scatterplot Smoothing)-smoothed trend. The red shaded area denotes the 95% bootstrap confidence band for the fitted trend. The vertical green dotted line indicates the bootstrap-derived median threshold (24.0 h), and the green shaded vertical band represents the 95% bootstrap confidence interval (23.0–27.0 h) for this threshold.

## 4 Discussion

This study developed an explainable model for predicting postoperative pulmonary infection in patients undergoing aSAH clipping and explored a potentially modifiable, time-sensitive care-process signal related to EN initiation timing. Among the evaluated algorithms, the Naive Bayes model demonstrated the best discriminative performance (AUC = 0.811) and showed favorable clinical utility across a wide range of threshold probabilities (9). Importantly, the integration of SHAP enabled transparent interpretation of both global and individual risk factors, enhancing the clinical applicability of the model. Compared with previously reported machine learning models for predicting pneumonia in aSAH patients (AUC typically ranging from 0.72 to 0.78), our model shows improved predictive performance, while maintaining interpretability through SHAP analysis (15–17).

Our findings suggest that PPI risk is determined by a combination of non-modifiable baseline characteristics and modifiable care-related factors. Stroke history and neurological severity (e.g., GCS score and Hunt–Hess grade) were identified as major predictors (18, 19), which is consistent with previous literature indicating that impaired consciousness and neurological dysfunction increase the risk of aspiration and pulmonary complications (20–22). These variables should therefore be regarded as “risk anchors” to trigger early preventive strategies rather than as direct intervention targets.

The primary model also identified several care-process-related variables associated with PPI predictions, including mechanical ventilation, nasogastric tube use, and EN initiation status by the 24-h landmark. These variables may help identify patients who warrant intensified preventive care; however, their predictive contributions should not be interpreted as causal effects. These factors represent an interconnected clinical pathway involving airway instrumentation, aspiration risk, and metabolic stress. Evidence from a large meta-analysis indicates that ventilator care bundles—particularly head-of-bed elevation, oral care, and early weaning strategies—can significantly reduce ventilator-associated pneumonia (VAP) incidence (OR≈0.42) and shorten ventilation duration and hospital stay (23, 24). From a systems perspective, these findings reinforce that PPI is not merely a consequence of disease severity, but a potentially preventable complication influenced by modifiable clinical practices. Our findings further support the concept that PPI is not solely determined by disease severity, but is also strongly influenced by modifiable care processes (23). Recent studies have also demonstrated the feasibility of integrating machine learning into nutritional and nursing decision-making in ICU settings, highlighting its potential to guide individualized care (25, 26).

From a nursing perspective, aspiration prevention is a critical component of care. Enteral feeding, while essential, is associated with a substantial risk of aspiration pneumonia, with reported incidence ranging widely in critically ill populations (20, 27). Nasogastric tube use may further increase reflux and microaspiration risk (27), especially in patients with impaired consciousness. Evidence-based strategies for aspiration prevention include maintaining head-of-bed elevation (30–45°), optimizing oral hygiene, minimizing sedation, and implementing early dysphagia screening (20, 28). For patients with persistent feeding intolerance or high aspiration risk, escalation to post-pyloric feeding may be considered, as it has been associated with reduced pneumonia risk in critically ill patients (29).

In the secondary exploratory analysis, continuous EN initiation timing showed a nonlinear association with model output. The LOWESS-smoothed SHAP trajectory crossed the zero baseline in the approximately 24–25-h region, and bootstrap resampling suggested internal stability in the estimated location of this model-derived inflection point. Current critical-care guidelines generally recommend early EN, although the precise timing of initiation remains incompletely defined and has varied across studies and clinical settings (7, 30–32). Importantly, however, a SHAP zero-crossing reflects a change in the direction of a feature's contribution relative to the model baseline and should not be interpreted as a clinically validated intervention threshold or optimal treatment time.

The observed association between EN initiation timing and PPI should also not be interpreted as evidence that delayed EN independently causes pulmonary infection. In routine clinical practice, EN initiation may be influenced by neurological status, hemodynamic stability, gastrointestinal tolerance, respiratory stability, and the overall complexity of the postoperative course. Consequently, delayed EN may partly function as a marker of underlying illness severity or clinical instability. Nevertheless, there is biological plausibility for a relationship between nutritional support and pulmonary outcomes. Experimental and clinical evidence has highlighted bidirectional interactions along the gut–lung axis (32, 33).while preservation of gastrointestinal barrier integrity and function represents an important rationale for enteral feeding in critically ill patients (34, 35). Previous evidence also suggests potential benefits of early enteral nutrition in critically ill populations (31, 32). These considerations provide biological context for the observed association but do not establish that the approximately 24–25-h model-derived inflection point represents a causal or clinically actionable cutoff. The EN-related findings should therefore be considered associative and hypothesis-generating and require prospective validation.

Therefore, our findings challenge the conventional generic “early” EN protocols and propose a shift toward a more precise, timesensitive nutritional intervention strategy. While the mathematical inflection point was observed around 25.0 h, the bootstrap 95% confidence interval (23.0–27.0 h) indicates that the protective effect is not confined to a single hour. From a clinical practice perspective, these data strongly suggest that initiating EN within 24 h of surgery represents a safe and effective window to mitigate PPI risk. We propose that clinicians prioritize early EN initiation (ideally < 24 h) rather than targeting an exact hour, as the biological benefits of maintaining gut mucosal integrity are most pronounced when nutritional support is not delayed into the second postoperative day. However, given the exploratory nature of this secondary analysis, this temporal signal should be interpreted with caution and requires prospective validation before clinical implementation.

These findings support a structured, risk-adapted nursing strategy for PPI prevention in aSAH patients. High-risk individuals identified by the model may benefit from: (1) implementation of ventilator care bundles (e.g., head-of-bed elevation, oral care, and sedation optimization); (2) strict aspiration prevention, including early dysphagia screening and positioning; (3) minimization of airway device exposure where feasible; and (4) timely review of EN initiation and nutritional support pathways. In clinical practice, such a model could potentially be used as a risk-stratification aid to prompt intensified preventive care, including ventilator bundle adherence, aspiration prevention, dysphagia screening, and earlier review of nutritional support pathways.

## 5 Limitations

Several limitations should be acknowledged. First, this was a single-center retrospective study, and the validation cohort represented temporal rather than multicenter external validation. Second, although the primary prediction model was restricted to information available at the prespecified 24-h landmark to minimize temporal information leakage, continuous EN initiation timing was evaluated separately in a secondary exploratory analysis. The observed 24–25-h inflection region was not based on a prespecified hypothesis and, despite its internal stability in bootstrap analysis, should be regarded as hypothesis-generating rather than a clinically validated cutoff. Third, detailed artery-level aneurysm locations were not consistently available, precluding reliable site-specific analyses. Future studies should therefore focus on prospective multicenter validation and dynamic prediction using time-dependent clinical information.

## 6 Conclusion

This study developed and temporally validated an explainable machine-learning model for predicting postoperative pulmonary infection after microsurgical clipping for aneurysmal subarachnoid hemorrhage. The Naive Bayes model achieved the best overall performance in the validation cohort, and SHAP analysis provided clinically interpretable explanations of both non-modifiable risk anchors and potentially modifiable care-process factors. A secondary exploratory analysis of continuous enteral nutrition initiation timing showed a nonlinear model-derived pattern, with an estimated inflection region around 24–25 h.

These findings suggest that explainable machine-learning models may support early risk stratification and risk-adapted nursing prevention strategies in patients with aneurysmal subarachnoid hemorrhage. The enteral nutrition timing result should be regarded as exploratory and requires prospective multicenter validation before being translated into clinical practice.

## Data Availability

The raw data supporting the conclusions of this article will be made available by the authors, without undue reservation.
